# A comparison of field portable X-ray fluorescence (FP XRF) and inductively coupled plasma atomic emission spectroscopy (ICP-AES) methods for analysis of metals on surface dust

**DOI:** 10.21203/rs.3.rs-3854350/v1

**Published:** 2024-01-15

**Authors:** Cara O’Donnell, Daniel Autenrieth, Raja Nagisetty

**Affiliations:** 1 Montana Technological University, Department of Safety, Health, and Industrial Hygiene, Butte, Montana, USA; 2 Montana Technological University, Department of Environmental Engineering, Butte, Montana, USA

**Keywords:** lead, correction factor, dust wipes, level of agreement, Superfund

## Abstract

The traditional method for sampling for lead on surfaces uses inductively coupled plasma atomic emission spectroscopy (ICP-AES) to analyze the concentration of lead and other metals on surfaces. This type of analysis is time consuming and costly. Field portable X-ray fluorescence (FP XRF) is another analysis method that is not as accurate as traditional laboratory methods but is more cost efficient and has a turnaround time of less than an hour. The primary goal of this study is to find the best method to increase the level of agreement between the ICP-AES concentrations and the FP XRF concentrations when analyzing lead concentrations on surface wipes. Inverse regression and ratio of the means correction factors were analyzed to try to improve the prediction of ICP-AES concentrations using FP XRF results. Fifty-seven dust wipe samples were analyzed using a split-half design. Half of the samples were used to create the correction factor and the other half were used to test the level of agreement. Linear regression and Bland -Altman plots were used to determine the correction factor that provided the highest level of agreement. A ratio of the means correction factor was determined to be the most appropriate.

## Introduction

This study is a part of a larger body of research which addressed the relationship between environmental health perceptions of members of the public in the Butte, MT Superfund community compared to the objectively measured environmental health exposures. In an effort to reduce the disconnect between public perceptions and measured exposures, a “little science” approach has been proposed, where members of the public measure and analyze their own exposures.

Butte, MT is home to the largest superfund community in the United States. Years of mining and smelting in the area lead to this designation (Nagisetty et al., 2020). One of the main concerns of this waste is heavy metal contamination. In the community of Butte, MT mining continues near residential areas. Hailer et al. (2017) studied the levels of heavy metals in hair of residents and then compared those levels to that of residents in a nearby community with no history of mining. Butte residents had statistically higher (α = 0.05) concentrations of Al, As, Cd, Cu, Mn, Mo, And U in their hair samples. Butte MT also had elevated levels of Cu, Zn, and Pb in Butte soil samples when compared to national standards (Hailer et al., 2017).

A thesis published in 1993 analyzed blood lead levels in Butte MT and found that “children living in pre-1950-homes had a statistically significant (P<0.01) higher mean blood lead concentration (1.6 ug/dL) than those living in post-1950 homes” (Canty, 1993, p.ii). A pooled analysis done by Lanphear et al. demonstrated that “lead contaminated household dust is the major source of exposure to lead in children with elevated blood lead levels” (1993, p.58). In 2012 the Centers for Disease Control and Prevention implemented a reference value of 3.5 micrograms per deciliter (ug/dL) blood lead level in children (CDC, 2022). This value represents the 97.5^th^ percentile of blood lead levels in children 1–5 years old. So, children that exceed the level of 3.5ug/dL blood lead level are in the top 2.5%. This replaced the previous level of concern of 10 ug/dL. However, blood lead levels below 10 ug/dL are harmful and are inversely associated with children’s IQ scores (Canfield et al., 2003). It is important that children with elevated blood lead levels receive services to decrease these levels because along with the effects on IQ, elevated blood lead levels can also cause cardiovascular, immunological, and endocrine effects (CDC,2022).

In addition to screening levels for blood lead, clearance levels for lead on surfaces were also updated in 2020 (EPA, 2022). EPA’s clearance levels are the level in which abatement work is considered successful. It demonstrates that the abatement effectively eliminated the lead hazards in residential paint, dust, and soil. The previous standards were 40μg/ft^2^ for floor dust and 250 μg/ft^2^ for windowsill dust. The updated standards are much more stringent at 10 μg/ft^2^ and 100 μg/ft^2^ for floor and windowsill dust, respectively (EPA, 2022). These standards now align with HUD Guidelines for controlling lead-based paint hazards in residential housing and other facilities. The goal of the reduced lead standards was to “better protect American children from the dangers of lead” (EPA, 2022). Lead based paint hazards in homes is still very prevalent in the Unites States, especially in lower income communities (Jacobs et al., 2002).

It is essential to have accurate and reliable results when determining the concentration of metals on surface wipe samples. These surface wipe samples will determine whether homes in the Butte, MT community have increased levels of lead and other heavy metals in them and if action needs to be taken to ensure the health of the inhabitants. The traditional method for determining metal concentrations on surface wipes is a laboratory-based method called inductively coupled plasma atomic emission spectroscopy (ICP-AES). ICP-AES is a spectral method that can determine the composition of elements in a sample and quantify those elements. It uses high energy plasma to emit photons, this excites the electrons which then try to move to a lower energy level. When the electrons do this, the excess energy is released as light. The element present is determined by the wavelength of the light. The intensity of the signal compared to a calibration curve to determine the concentration of each particular element (Murray et al., 2000). The ICP-AES analytical process is traditional in that samples must be sent to a lab, analyzed, and then reported back to the customer. This analytical process typically takes a few weeks to receive the results and it can be costly. However, it is desirable because its low limits of detection and ability to detect a wide range of metals.

Field portable X-ray fluorescence (FP-XRF) is a non-destructive method which allows for a relatively short analytical time and is more cost efficient than ICP-AES. The FP XRF device uses an X-ray source (Cd40) to cause the electrons in the sample to increase in energy move to higher orbitals. This causes the atoms to become unstable and electrons will return back to inner orbitals. This results in the release of x-rays that are characteristic for specific elements and these x-rays are analyzed by the FP XRF device. This energy is known as x-ray fluorescence. The device reads the intensity and energy which relates to a certain element and quantity of that element. “For example, if lead (Pb) is present in an object, an XRF signal will be detected at 10.55 and 12.61 keV, and its quantity can be determined by plotting the energy (E) vs. intensity (I).” (Blondel, 2020)

FP XRF devices can be used for air filters, bulk samples of soil, positive material identification, dust wipes, and lead paint samples (NITON, 2004). Sterling et al. (2000) evaluated the agreement between FP XRF dust wipe analysis for lead and a traditional laboratory method, flame atomic absorption spectrophotometry (FAAS). They found the paired data to be highly correlated (R^2^ = 0.89) between the two methods. However, paint chips in the dust wipe samples can confound the results (Sterling et al., 2000). When comparing FP-XRF analysis and ICP-AES analysis for bulk samples of soil around homes with lead-based paint Binstock et al. (2009) found no statistical difference between the two methods in soil with particle sizes less than 250 μm. When comparing ICP and XRF for lead on surface wipes, Harper et al. (2002) concluded that the XRF underestimated the concentrations but still found strong liner relationships between the two methods.

### Objectives

The primary goal of this study is to evaluate the level of agreement between lead concentrations in dust wipe samples analyzed via FP XRF in comparison with the results obtained from laboratory ICP-AES analysis. First, a calibration factor for lead on thin film samples will be developed using known concentrations of lead on thin film samples and applied to the FP XRF device. Then correction factors will be established to predict ICP-AES concentrations given the FP XRF concentrations. Finally, a level of agreement between the corrected FP XRF concentrations and the ICP - AES concentrations will be evaluated to determine the most accurate correction factor.

The secondary goal of this study is to evaluate the level of agreement between manganese, molybdenum, arsenic, and copper concentrations in dust wipe samples analyzed via FP XRF in comparison with the results obtained from laboratory ICP-AES analysis.

## Methods and materials

### Surface Sampling

**Figure 1:**
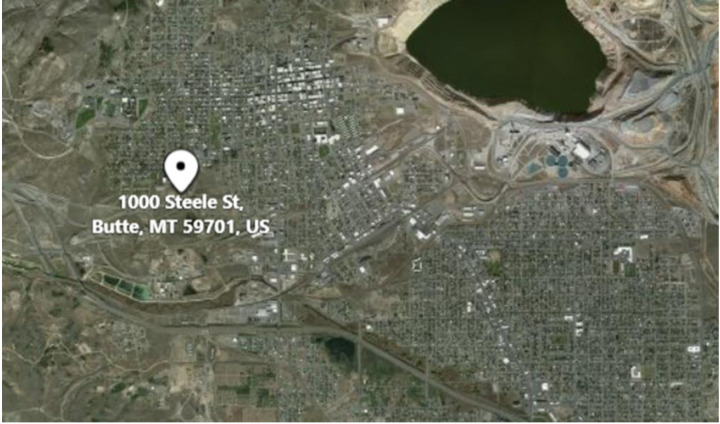
Location of soil collected for this study at the Superfund Site in Butte MT

Fifty-seven samples were collected using a modified version of NIOSH method 9102 – elements on wipes (NIOSH,2003). An aerosol chamber was utilized to aerosolize mine tailings collected in Butte, MT, collected at coordinates (46.0042, −112.5511), Figure 1. This tailings pile in Butte MT has been demonstrated to contain high levels of lead when bulk samples of the soil are analyzed via the FP XRF. The aerosol chamber allows bulk particulate matter fines to be suspended in air and once airborne, the dispersed aerosols can be captured using traditional integrated sampling media and methods or with direct reading methods. The chamber uses a combination of compressed air and a magnetic stirring plate to aerosolize particulate matter fines from a beaker. The aerosolized particulate matter is then transported to the top of the chamber via. Tigon tubing where it is dispersed through a plenum on the top of the chamber box. After distribution, the settling velocities of the suspended particulate matter determines the amount of time that the aerosol remains suspended. Air is drawn through a high-efficiency particulate absorbing (HEPA) filter and maintains a slight negative pressure within the chamber.

The bulk tailings were sieved down to 150 microns and smaller. Fifteen mg of the sieved tailings were then placed in a clean beaker and dispersed in the aerosol chamber for 15 minutes. Wipe samples were then collected using 10 cm × 10 cm templates to ensure consistent surface area measured from the floor of the chamber. SKC GhostWipes were used for the surface sampling as they meet all ASTM E1792 specifications for sampling lead and other surface metals (ASTM, 2011). The wipe sample was taken by swiping the 10 cm × 10 cm template up and down, followed by folding the wipe with contaminants inside and repeating the wipe side to side and folding again (Brookhaven National Laboratory, 2017). After each sampling, the wipes were dried overnight in the lab. Drying wet wipes prior to FP XRF analysis has been demonstrated to improve the accuracy and precision of the XRF device along with decreasing the potential for scattering of the x-rays (Bayne et al., 2003, p.2).

The surface wipe samples were analyzed initially using a ThermoFisher Scientific field portable X-ray fluorescence device (XLP-703) in dust wipe mode. Per the manufacturer’s instructions, wipes were analyzed in separate positions. First at the number one position for 15 source seconds then the sample was placed at the number 2 position and analyzed for another 15 source seconds. The wipe was rotated 180 degrees and placed at the number 1 position again for a third 15 source second measurement and the final reading was in the number 2 position for 15 source seconds (Bayne et al., 2003, p.3). The measurements were averaged, and metal concentrations were represented in μg/sample. The x-ray tube on the XLT 700 spectrum analyzer was over 2 years old so the time to complete each individual measurement was approximately 15 minutes. The decay and half lifetime corrections are not factors for x-ray tubes (Bayne et al., 2003, p.3). This increased time does not affect the results however it does affect the convenience when using this device as a real time measurement tool for the public to utilize. After the surface wipe samples were analyzed using the FP XRF device, the samples were sent to ALS Laboratory in Salt Lake City, an AIHA accredited laboratory, to be analyzed using the NIOSH method 9102, Issue 1: Elements on Wipes (NIOSH,2003). The NIOSH method uses inductively coupled plasma atomic emission spectroscopy (ICP-AES) for analysis. ICP-AES analysis was performed for a full metals panel which includes the metals discussed in this study (Pb, Mo, Mn, As, Cu).

### Statistical Analysis

#### FP XRF Calibration

In order to accurately compare the concentrations between ICP-AES and FP XRF, a calibration factor was created for the XRF device. Lead Paint Standards from ThermoFisher Scientific were used for the calibration factor. The five standards were analyzed three times for ten source seconds each, along with the blank. The known metal concentrations were plotted against the measured concentrations reported by the FP XRF. A liner regression equation was produced using this relationship. The Lead Paint Standard calibration values were treated as the predictor (X) variable and the concentrations measured from the XRF were considered the response (Y) variable. The inverse regression equation for lead was used to correct the concentrations from the XRF thus creating the calibrated FP XRF concentrations.

#### Comparison of ICP-AES Concentrations to Calibrated FP XRF Concentrations

A Spearman correlational analysis was performed between the ICP – AES concentrations and the calibrated FP XRF concentrations for lead on surface wipes in order to assess the first research objective of developing a calibration factor for the FP XRF device and the corelating strength of associations. The contaminant concentrations were not normally distributed when using the Anderson-Darling test for normality. Log_10_ transformations of the lead data was applied, and it was determined that they were not normally distributed.

Regarding the next research objective of determining calibration factors to apply to the XRF results, two calibration factors were analyzed. First the paired ICP – AES results and their corresponding XRF results were randomly assigned a group. The first half was deemed the “model set” and were used to create the correction factors. The second half of the data was deemed the “test set” and was used to test the correction factors on. Similar to the calibration factor, an inverse regression correction factor was developed using the model set of data. The ICP – AES values were treated as the predictor (X) variable and the calibrated FP XRF values were treated as the response (Y) variable. The inverse regression equation developed from this relationship was used as the first correction factor/equation. This equation was used on the test set with the goal of predicting the ICP – AES values. The second correction factor/equation developed using the model set of the data was a ratio of the means. This was determined by the mean of the ICP – AES concentrations divided by the mean of the FP XRF concentrations. This ratio was then applied to the test set of XRF concentrations.

The level of agreement between the corrected FP XRF concentrations and the ICP - AES concentrations were evaluated to determine the most accurate correction factor. To determine the level of agreement the ratio corrected XRF data, inverse regression corrected XRF data, uncorrected XRF data, and the ICP – AES data were compared using box and whisker plots. To determine the level of agreement between the ICP – AES lead concentrations and the corrected XRF concentrations Bland- Altman plots were developed.

Minitab Statistical Software version 21 was used for all statistical analyses. (Version 21, State College, PA).

## Results and Discussion

For the calibration factor, five lead paint standards were analyzed via. the FP XRF and plotted against the known concentration to determine the calibration factor. The percent difference between the FP XRF concentrations and the known standard concentrations differ. The table below shows the percent difference between the standard concentrations and the FP XRF results. In general, as the known standard concentrations increase, the percent difference between the standard concentrations and the FP XRF results also increase. So, without a calibration factor, the results on the higher end are not going to be as accurate.

**Figure 3:**
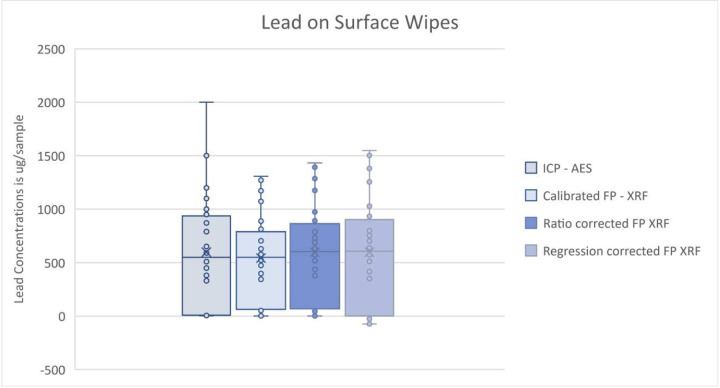
Box and whisker plots to compare ICP-AES, Calibrated FP XRF, Ratio corrected FP XRF and Regression corrected FP XRF data

A total of 57 wipe samples were collected using the aerosol chamber. For lead, the concentrations ranged from 3.4 – 2000.0 ug/sample and 4.9 – 1369.7 ug/sample from the ICP-AES analysis and FP XRF analysis, respectively. Of the 57 samples, all were above the Limit of Detection for lead (0.50 μg/sample) for the ICP – AES analysis and 52 were above the LOD for the FP XRF. The 5 FP XRF samples below the LOD were treated as censored data and were included in the paired data results as censored data (LOD/√2) per Janicak (2007). To address the first objective of the research, correlational strength was determined between the ICP – AES data and its corresponding calibrated FP XRF values, as shown in Figure 3. The ICP-AES and calibrated FP XRF lead concentrations demonstrated a strong positive correlation with a Spearman coefficient of 0.885 and an R^2^ value of 0.7608 during regression analysis. Harper et al. (2002) also found strong linear relationships between XRF and ICP methods for lead on surface wipes with an R^2^ value of 0.973.

Table 2 below summarizes the descriptive statistics for the calibrated FP XRF test data, ICP – AES test data, and the corrected (ratio and inverse regression) FP XRF data. The arithmetic mean for lead concentrations analyzed by ICP-AES was 598.4 ug/sample and 543.6 ug/sample for the uncorrected, calibrated FP XRF concentrations. The arithmetic means for the ratio corrected and regression corrected concentrations are 595.8 ug/sample and 598.5 ug/sample respectively.

**Figure 4:**
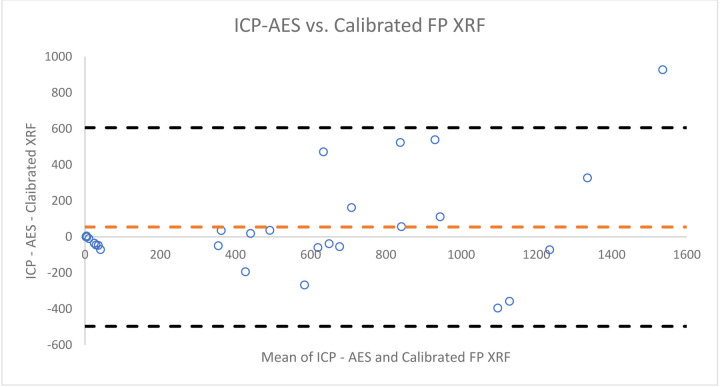
Bland-Altman Plot of the differences between lead concentrations measured by ICP - AES and calibrated FP XRF data vs. the mean of the two concentrations

Table 3 displays the correction factor equations along with the calibration equation determined from Lead Paint Standards for lead on surface wipes. A box and whisker plot for lead is shown in Figure 4.

**Figure 5:**
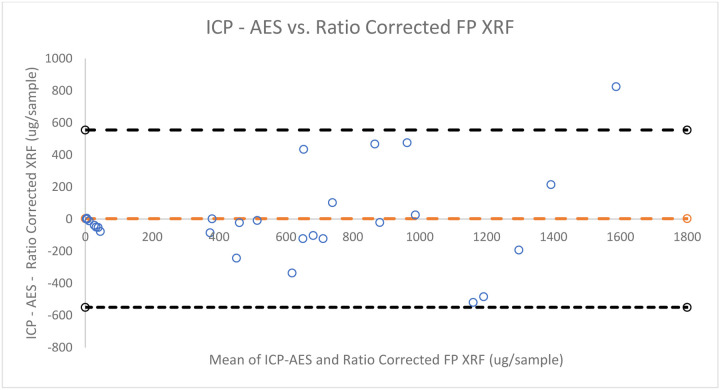
Bland-Altman Plot of the differences between lead concentrations measured by ICP - AES and ratio corrected FP XRF data vs. the mean of the two concentrations

**Figure 6:**
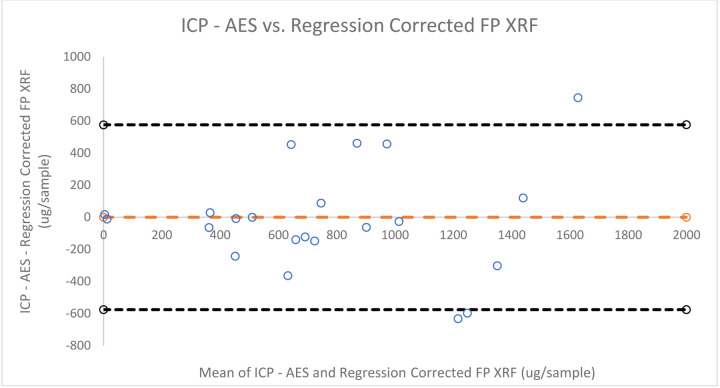
Bland-Altman Plot of the differences between lead concentrations measured by ICP - AES and regression corrected FP XRF data vs. the mean of the two concentrations

**Figure 7:**
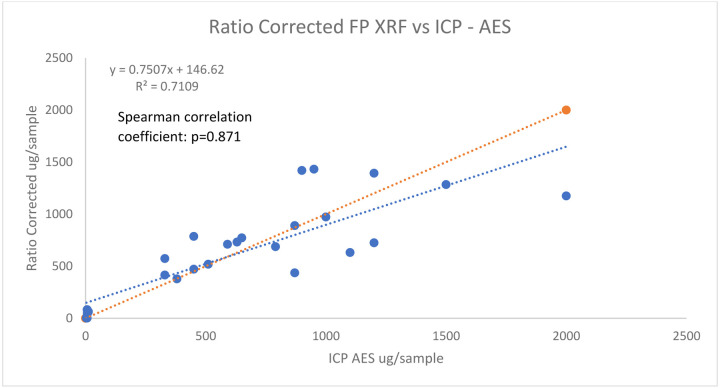
The association between ICP - AES concentrations and ratio corrected FP XRF concentrations for lead on surface wipes

Figures 5,6, and 7 provide qualitative Bland-Altman plots for uncorrected lead data, ratio corrected data, and regression corrected data respectively. The mean differences and 95% limits of agreement are shown in each plot. For lead the agreement interval width between ICP-AES and the calibrated FP XRF concentrations was 1,102.33. When comparing ICP-AES to the ratio corrected FP XRF concentrations using a Bland- Altman plot the agreement interval width is 1,104.77. And when comparing ICP-AES concentrations to regression corrected FP XRF concentrations the agreement interval width increased to 1,151.99.

The agreement interval width increased slightly after adding the ratio correction factor in comparison with the PF XRF results without a correction factor.

**Figure 8:**
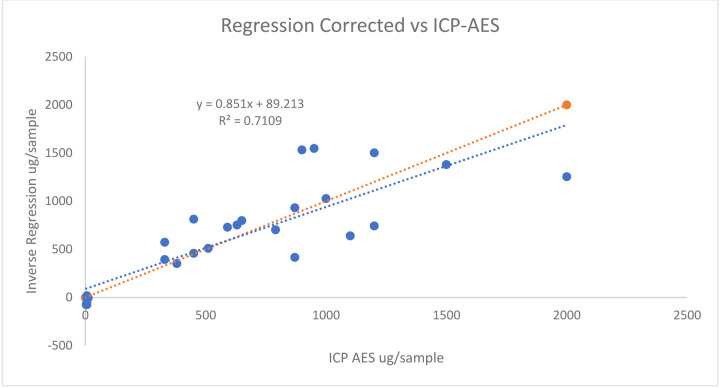
The association between ICP - AES concentrations and regression corrected FP XRF concentrations for lead on surface wipes

**Figure 9:**
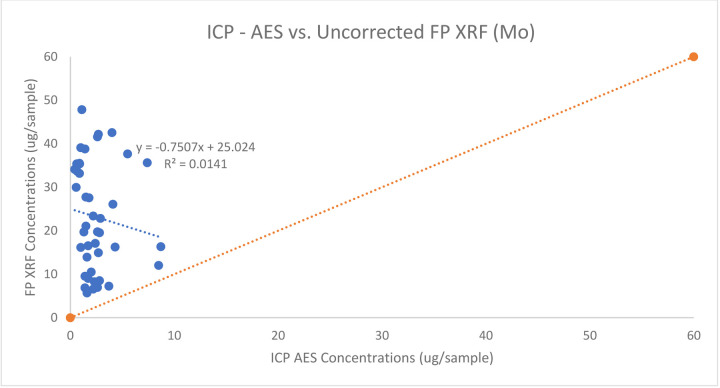
The association between ICP AES vs FP XRF Concentrations for Mo on surface wipes

Figures 8 and 9 displays the correlational strength between the ICP-AES concentrations and the ratio corrected and inverse regression corrected FP-XRF concentrations.

The table below displays the mean differences between the ICP-AES reference concentrations compared to the three different FP XRF concentrations (calibrated, ratio corrected, and regression corrected). When applying a correction factor, the mean difference improves the agreement between the two analytical methods. With a mean difference of −0.1 for the regression corrected FP XRF data compared to the ICP-AES reference concentrations it would appear like the regression correction factor gives the best improvement. However, the regression correction factor creates negative values at the low concentrations which is impractical when determining the most accurate correction factor. The mean difference improves when applying the ratio correction factor in comparison to the uncorrected, calibrated FP XRF concentrations from 54.8 to 2.6. The ratio correction factor does not create any negative values which allows it to be a more practical option as a correction factor.

Of the 57 surface wipes analyzed, 23 of the wipes had detectable levels of arsenic via. ICP-AES analysis and zero of those surface wipes had detectable levels of arsenic when analyzed using the FP XRF. Due to this lack of detectable arsenic via. FP XRF analysis, it was not possible to have paired data or develop a correction factor for arsenic on surface wipes.

Of the surface wipes analyzed, all 57 had detectable concentrations of molybdenum via. ICP-AES and 30 were above the LOD for the FP XRF. The samples ranged from 0.42 – 12 μg/sample and 5.68 – 47.84 μg/sample for molybdenum from the ICP-AES analysis and FP XRF respectively. The samples 30 samples were paired and plotted against each other. The agreement between the ICP-AES and FP XRF values was poor with an (R^2^ = 0.0141) and therefore a meaningful correction factor was not able to be developed for molybdenum on dust wipes. The figure below shows the agreement between FP XRF concentrations and ICP AES concentrations for Mo on surface wipes.

Another metal of concern analyzed on the surface wipes is copper. All 57 of the wipes had detectable concentrations of copper when analyzed via. ICP-AES and 24 were able the LOD when analyzed using the FP XRF. The samples ranged from 3.6 – 51.0 μg/sample from the ICP - AES analysis and 7.35 – 95.65 μg/sample from the FP XRF analysis. The 24 samples were paired and plotted against each other and the agreement between the two concentrations, similar to molybdenum, was poor (R^2^ =.088) and a correction factor was not able to be developed for copper. The figure below shows the agreement between FP XRF concentrations and ICP AES concentrations for Cu on surface wipes.

The final metal of concern analyzed on the surface wipes was manganese. Again, all 57 of the wipes had detectable concentrations of manganese when analyzed via. ICP – AES. Thirteen of the samples had detectable concentrations of manganese when analyzed via. FP - XRF. Due to there not being enough paired concentrations, a correction factor was not able to be developed for manganese. The figure below shows the paired data concentrations for Mn and how significantly the FP XRF overestimates exposures. These findings do not agree with a previous thesis from Montana Technological University that evaluated the agreement between FP XRF technology and ICP AES for manganese air concentrations. Tyler Davis, 2019 found FP XRF to be a viable option for quantifying manganese concentrations in occupational environments.

## Limitations

To properly compare FP XRF concentrations with ICP – AES reference concentrations, a calibration factor needs to be applied to the ray FP XRF data. For the metals analyzed other than lead (Cu, Mn, As, Mo), no standards were available to properly calibrate the FP XRF results. Therefore, the paired values for copper, arsenic, manganese, and molybdenum could not be adequately compared.

## Conclusions

This study determined that when measuring for lead concentrations in surface dust, the ratio of the means correction factor provides the best correction factor for lead on surface wipes when analyzed using a FP XRF device. This factor has the narrowest width of agreement between the two correction factors and improved the mean difference between the ICP AES concentrations and the FP XRF concentrations using a paired-t test. It also is the most practical correction when applying it in the Butte MT community as it did not result in any negative values, compared to the inverse regression correction factor which resulted in negative concentrations at the lower levels.

Due to the poor agreement between FP – XRF and ICP-AES concentrations for arsenic, manganese, copper, and molybdenum, and the high number of samples below the limit of detection when detectable limits are found via. ICP – AES analysis, when sampling for these metals in dust on surface wipes, without a correction factor applied, traditional laboratory methods such as ICP-AES analysis is the best option to get accurate results of the concentrations. This study revealed the FP XRF method was not reliable for other metal analysis without a calibration factor. Future studies could be beneficial to understand the agreement between PF XRF and ICP-AES for metals other than lead if calibration standards for those metals are used.

FP XRF technology is a practical and cost-effective option to use when analyzing for lead in surface dust. It is an attractive method when analyzing for lead, however if more than lead is being analyzed, ICP-AES technology can provide a more comprehensive understanding of the exposures.

FP XRF technology can be used as the “little science” approach when applied to the Butte, MT community. With the ratio calibration factor, the FP XRF can be used to increase community engagement in Butte MT by allowing residents to take their own dust samples in their residences and receive timely and cost-efficient results for surface dust lead concentrations in their homes.

This study demonstrated that adding a ratio of the means correction factor to calibrated FP XRF results for lead concentrations on surface wipes improves the accuracy of the device. The next step is to use calibration standards for other metals of concern to improve the agreement between ICP AES and FP XRF concentrations for metals other than lead. Then, ideally, correction factors could be applied to the FP XRF using the same method to improve the accuracy for multiple metal contaminants on surface wipes.

## Figures and Tables

**Figure 2:**
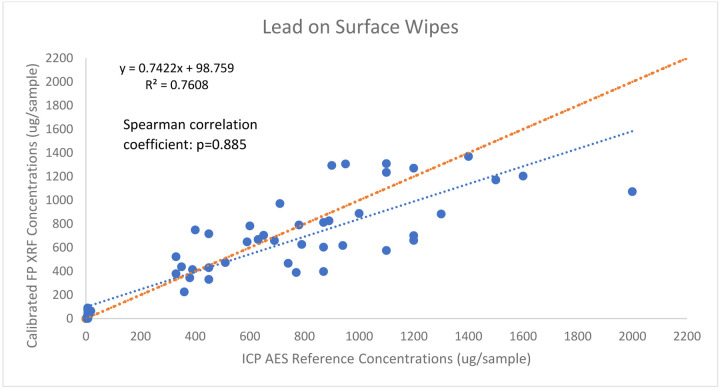
The association between lead (n=57) on surfaces obtained by ICP - AES and calibrated FP XRF

**Figure 10:**
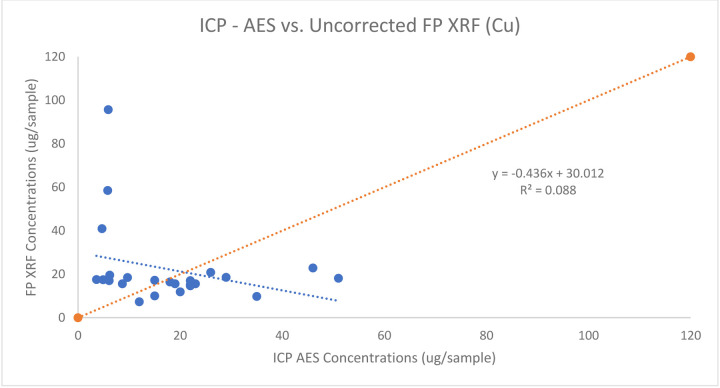
The association between ICP AES vs FP XRF Concentrations for Cu on surface wipes

**Table 1:** Percent difference between Lead Paint Calibration Standard and FP XRF Results

Percent difference between Lead Paint Calibration Standard and FP XRF Results		
Lead Paint Standard mg/cm^2^	FP XRF Results mg/cm^2^	Percent Difference
0.31	0.305	1.72
0.71	0.65	9.60
1.04	0.98	5.69
1.53	1.35	12.5
3.58	2.58	32.6

**Table 2:** Summarized statistics of lead concentrations (ug/sample) measured by ICP - AES and uncorrected and corrected XRF concentrations

Lead (μg/sample)								
Variable	Mean	SE Mean	StDev	Minimum	Q1	Median	Q3	Maximum
ICP - AES	598.4	98.7	522.1	3.4	8.6	550.0	937.5	2000.0
FP XRF Calibrated	543.6	80.2	424.1	1.5	62.9	549.9	788.8	1307.2
Ratio Corrected	595.8	87.9	464.9	1.6	69.0	602.7	864.5	1432.8
Regression Corrected	598.5	99.6	527.0	−75.2	1.2	606.3	903.1	1547.3

**Table 3:** Calibration and correction factors for lead on surface wipes

Calibration and Correction Factors - Lead		
Calibration Equation	Regression Correction Factor	Ratio Correction Factor
x=(y+0.195)/0.6809	x=(y+61.97)/0.8048	576.3/525.8 = 1.09

**Table 4:** Mean differences between ICP-AES reference concentrations and calibrated FP XRF, ratio corrected FP XRF, and regression corrected FP XRF concentrations

Comparisons with ICP-AES reference concentrations - Lead		
Method	Mean difference (95% CI)	P-value
Calibrated FP XRF	54.8 (−54.4, 163.8)	0.312
Ratio Corrected FP XRF	2.6 (−106.7, 111.8)	0.962
Regression Corrected FP XRF	−0.1(−114.0.113.9)	0.999

**Table 5:** Manganese results for ICP-AES and FP XRF

ICP AES Results (ug/sample) Mn	FP XRF (ug/sample) Mn
33	24.12
18	31.34
42	34.37
34	49.01
34	53.64
31	216.15
24	259.56
21	270.13
15	276.97
26	327.82
24	531.23
33	1270
13	2155
